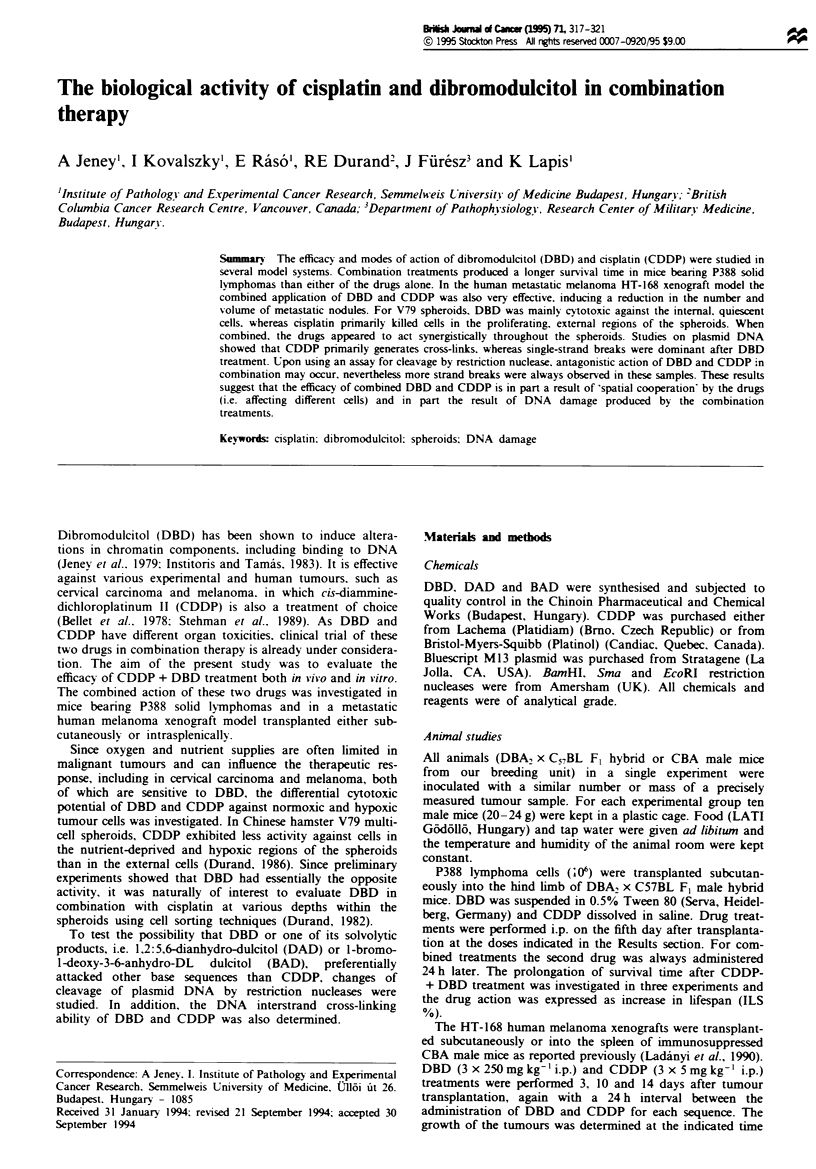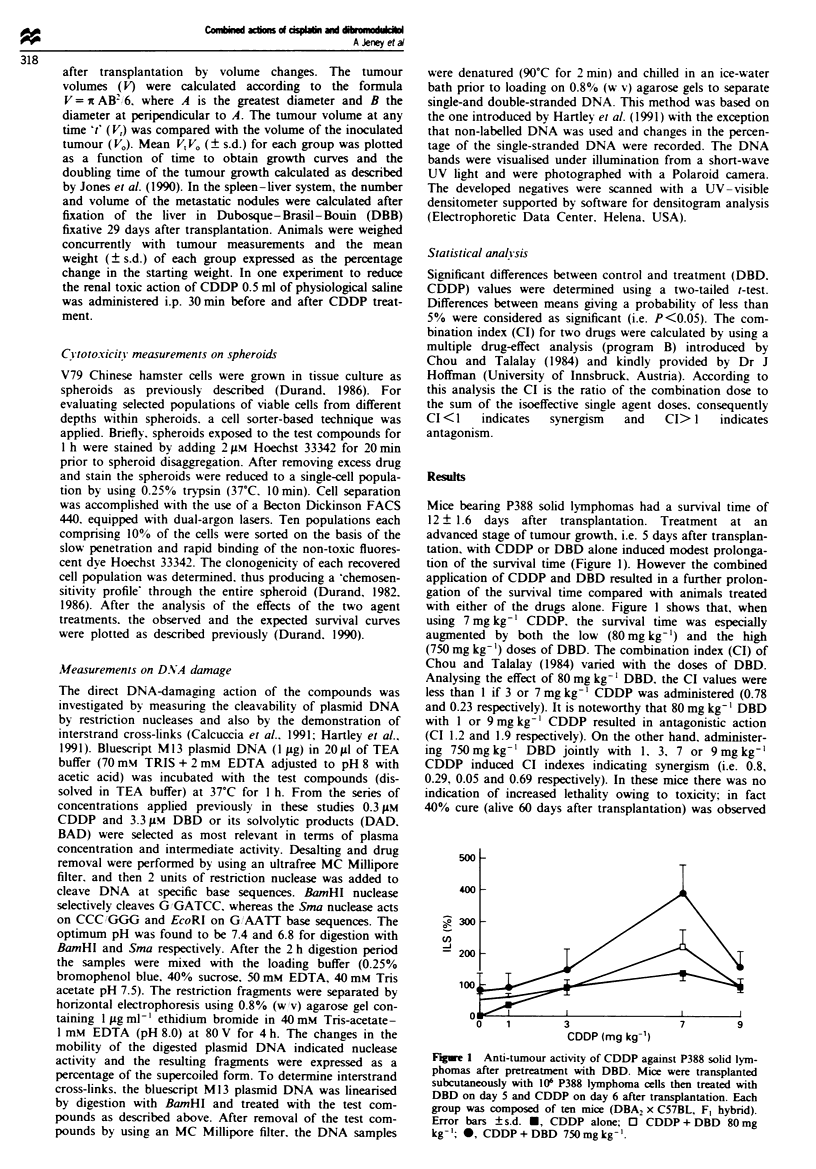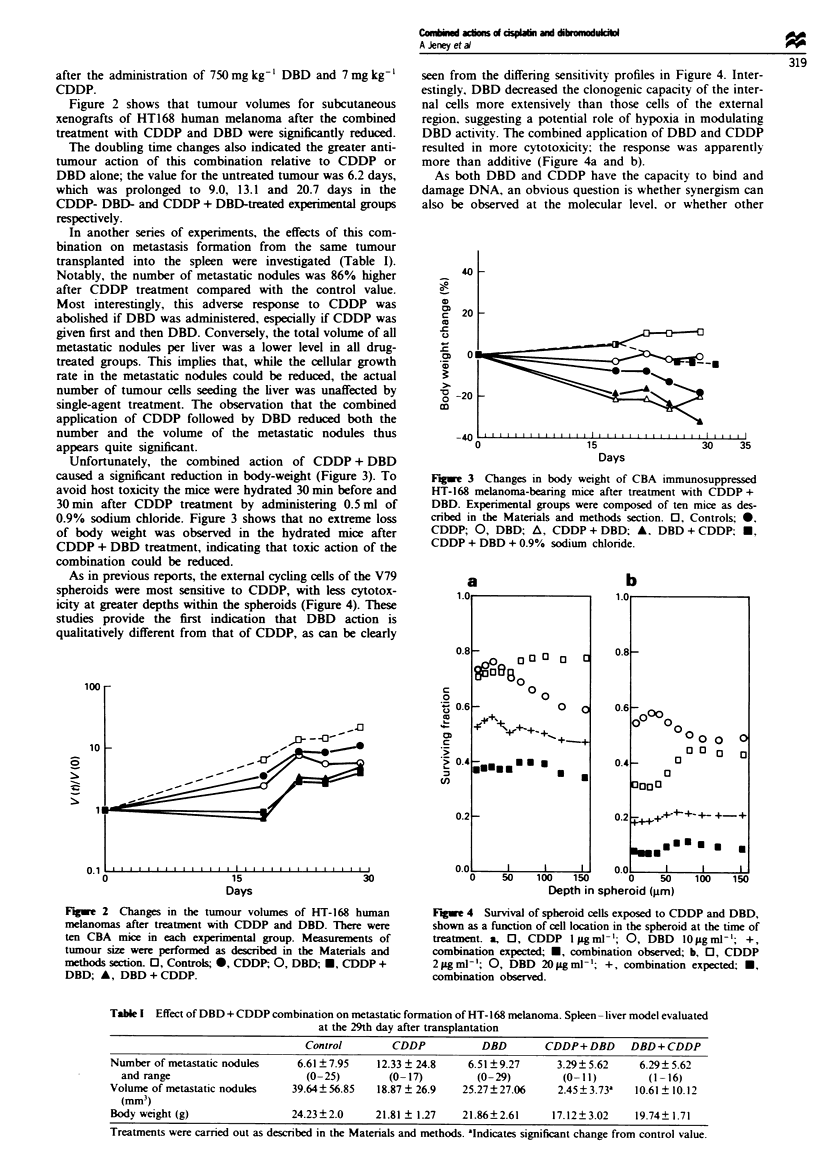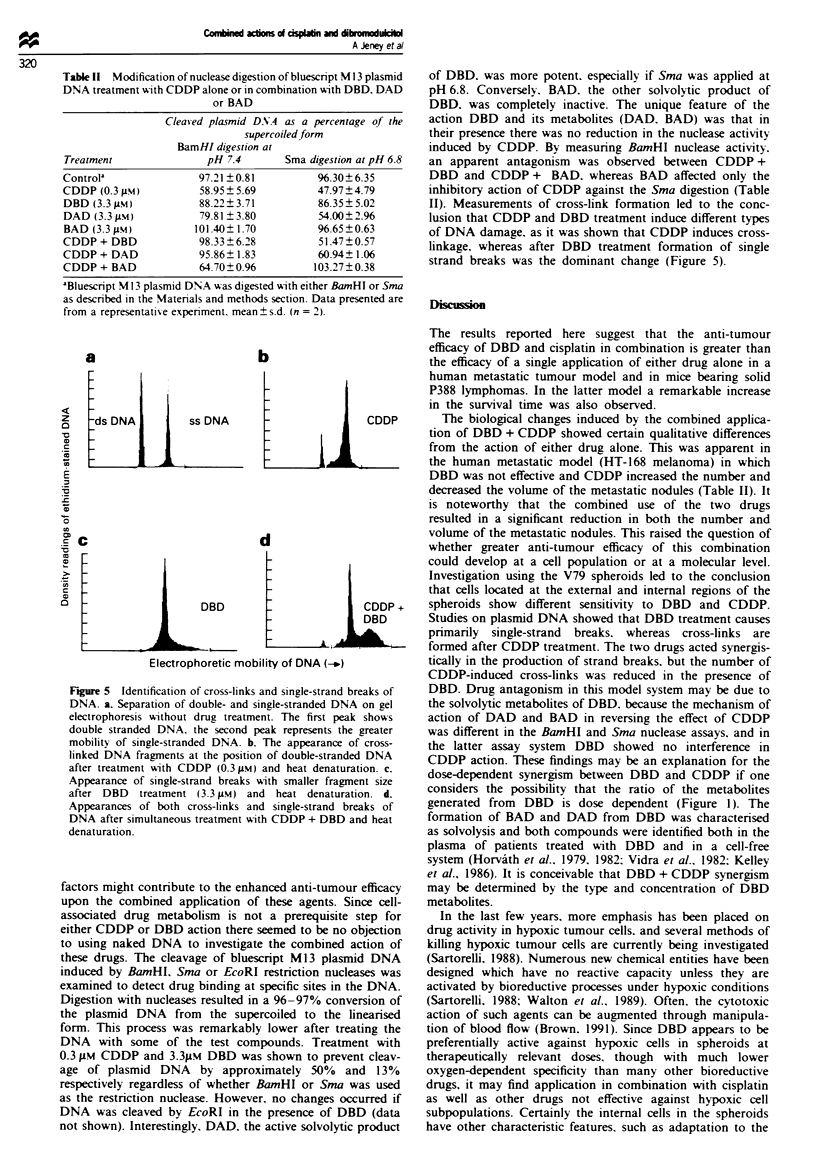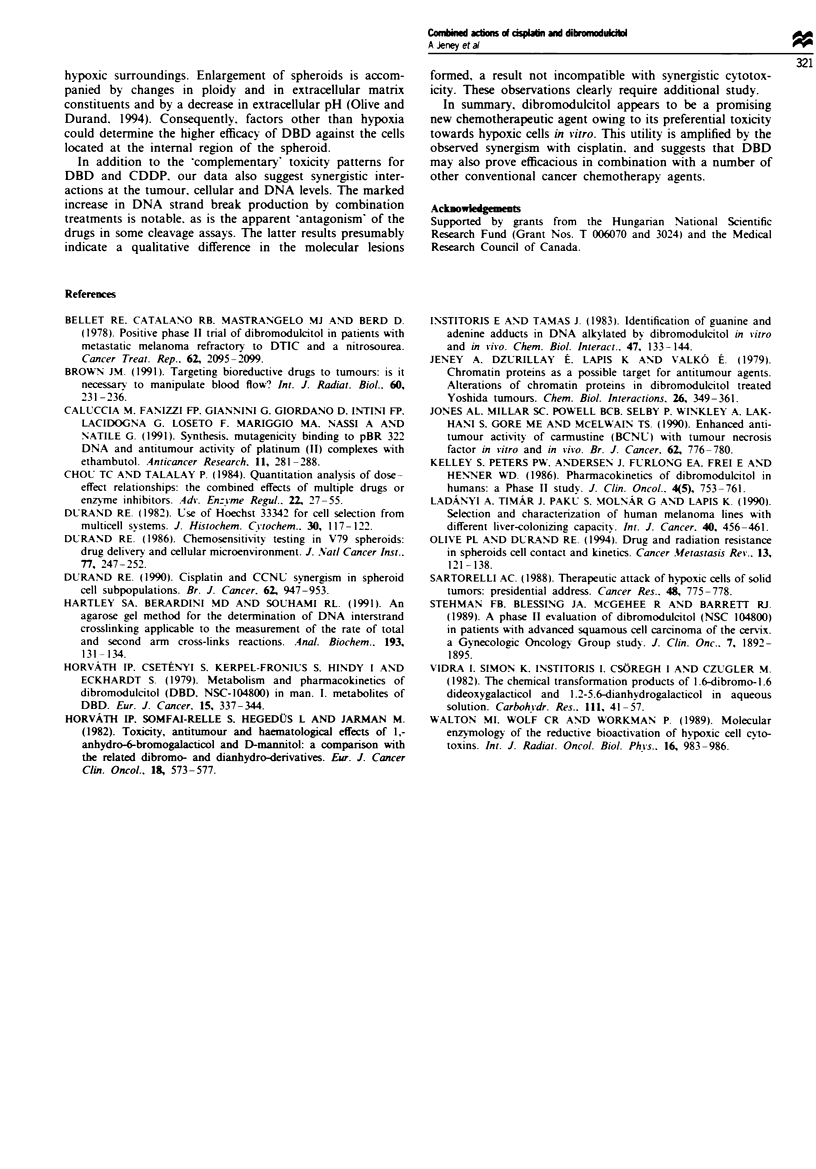# The biological activity of cisplatin and dibromodulcitol in combination therapy.

**DOI:** 10.1038/bjc.1995.63

**Published:** 1995-02

**Authors:** A. Jeney, I. Kovalszky, E. Rásó, R. E. Durand, J. Fürész, K. Lapis

**Affiliations:** Institute of Pathology and Experimental Cancer Research, Semmelweis University of Medicine Budapest, Hungary.

## Abstract

The efficacy and modes of action of dibromodulcitol (DBD) and cisplatin (CDDP) were studied in several model systems. Combination treatments produced a longer survival time in mice bearing P388 solid lymphomas than either of the drugs alone. In the human metastatic melanoma HT-168 xenograft model the combined application of DBD and CDDP was also very effective, inducing a reduction in the number and volume of metastatic nodules. For V79 spheroids, DBD was mainly cytotoxic against the internal, quiescent cells, whereas cisplatin primarily killed cells in the proliferating, external regions of the spheroids. When combined, the drugs appeared to act synergistically throughout the spheroids. Studies on plasmid DNA showed that CDDP primarily generates cross-links, whereas single-strand breaks were dominant after DBD treatment. Upon using an assay for cleavage by restriction nuclease, antagonistic action of DBD and CDDP in combination may occur, nevertheless more strand breaks were always observed in these samples. These results suggest that the efficacy of combined DBD and CDDP is in part a result of 'spatial cooperation' by the drugs (i.e. affecting different cells) and in part the result of DNA damage produced by the combination treatments.


British Journal of Cancer (1995) 71, 317-321

© 1995 Stockton Press All rights reserved 0007-0920/95 $9.00

### The biological activity of cisplatin and dibromodulcitol in combination therapy

A Jeney[1], I Kovalszky[1], E Rásó[1], RE Durand[2], J Fürész[3] and K Lapis[1]

[1]Institute of Pathology and Experimental Cancer Research, Semmelweis University of Medicine Budapest, Hungary; [2]British Columbia Cancer Research Centre, Vancouver, Canada; [3]Department of Pathophysiology, Research Center of Military Medicine, Budapest, Hungary.

**Summary** The efficacy and modes of action of dibromodulcitol (DBD) and cisplatin (CDDP) were studied in several model systems. Combination treatments produced a longer survival time in mice bearing P388 solid lymphomas than either of the drugs alone. In the human metastatic melanoma HT-168 xenograft model the combined application of DBD and CDDP was also very effective, inducing a reduction in the number and volume of metastatic nodules. For V79 spheroids, DBD was mainly cytotoxic against the internal, quiescent cells, whereas cisplatin primarily killed cells in the proliferating, external regions of the spheroids. When combined, the drugs appeared to act synergistically throughout the spheroids. Studies on plasmid DNA showed that CDDP primarily generates cross-links, whereas single-strand breaks were dominant after DBD treatment. Upon using an assay for cleavage by restriction nuclease, antagonistic action of DBD and CDDP in combination may occur, nevertheless more strand breaks were always observed in these samples. These results suggest that the efficacy of combined DBD and CDDP is in part a result of 'spatial cooperation' by the drugs (i.e. affecting different cells) and in part the result of DNA damage produced by the combination treatments.

**Keywords:** cisplatin; dibromodulcitol; spheroids; DNA damage

Dibromodulcitol (DBD) has been shown to induce alterations in chromatin components, including binding to DNA (Jeney *et al.*, 1979; Institoris and Tamás, 1983). It is effective against various experimental and human tumours, such as cervical carcinoma and melanoma, in which *cis*-diamminedichloroplatinum II (CDDP) is also a treatment of choice (Bellet *et al.*, 1978; Stehman *et al.*, 1989). As DBD and CDDP have different organ toxicities, clinical trial of these two drugs in combination therapy is already under consideration. The aim of the present study was to evaluate the efficacy of CDDP + DBD treatment both *in vivo* and *in vitro*. The combined action of these two drugs was investigated in mice bearing P388 solid lymphomas and in a metastatic human melanoma xenograft model transplanted either subcutaneously or intrasplenically.

Since oxygen and nutrient supplies are often limited in malignant tumours and can influence the therapeutic response, including in cervical carcinoma and melanoma, both of which are sensitive to DBD, the differential cytotoxic potential of DBD and CDDP against normoxic and hypoxic tumour cells was investigated. In Chinese hamster V79 multicell spheroids, CDDP exhibited less activity against cells in the nutrient-deprived and hypoxic regions of the spheroids than in the external cells (Durand, 1986). Since preliminary experiments showed that DBD had essentially the opposite activity, it was naturally of interest to evaluate DBD in combination with cisplatin at various depths within the spheroids using cell sorting techniques (Durand, 1982).

To test the possibility that DBD or one of its solvolytic products, i.e. 1,2:5,6-dianhydro-dulcitol (DAD) or 1-bromo-1-deoxy-3-6-anhydro-DL dulcitol (BAD), preferentially attacked other base sequences than CDDP, changes of cleavage of plasmid DNA by restriction nucleases were studied. In addition, the DNA interstrand cross-linking ability of DBD and CDDP was also determined.

Correspondence: A Jeney, I. Institute of Pathology and Experimental Cancer Research, Semmelweis University of Medicine, Üllői út 26, Budapest, Hungary - 1085
Received 31 January 1994; revised 21 September 1994; accepted 30 September 1994

### Materials and methods

### Chemicals

DBD, DAD and BAD were synthesised and subjected to quality control in the Chinoin Pharmaceutical and Chemical Works (Budapest, Hungary). CDDP was purchased either from Lachema (Platidiam) (Brno, Czech Republic) or from Bristol-Myers-Squibb (Platinol) (Candiac, Quebec, Canada). Bluescript M13 plasmid was purchased from Stratagene (La Jolla, CA, USA). *Bam*HI, *Sma* and *Eco*RI restriction nucleases were from Amersham (UK). All chemicals and reagents were of analytical grade.

### Animal studies

All animals ($DBA_2 \times C_{57}BL$ $F_1$ hybrid or CBA male mice from our breeding unit) in a single experiment were inoculated with a similar number or mass of a precisely measured tumour sample. For each experimental group ten male mice (20-24 g) were kept in a plastic cage. Food (LATI Gödöllő, Hungary) and tap water were given *ad libitum* and the temperature and humidity of the animal room were kept constant.

P388 lymphoma cells ($10^6$) were transplanted subcutaneously into the hind limb of $DBA_2 \times C57BL$ $F_1$ male hybrid mice. DBD was suspended in 0.5% Tween 80 (Serva, Heidelberg, Germany) and CDDP dissolved in saline. Drug treatments were performed i.p. on the fifth day after transplantation at the doses indicated in the Results section. For combined treatments the second drug was always administered 24 h later. The prolongation of survival time after CDDP + DBD treatment was investigated in three experiments and the drug action was expressed as increase in lifespan (ILS %).

The HT-168 human melanoma xenografts were transplanted subcutaneously or into the spleen of immunosuppressed CBA male mice as reported previously (Ladányi *et al.*, 1990). DBD ($3 \times 250$ mg $kg^{-1}$ i.p.) and CDDP ($3 \times 5$ mg $kg^{-1}$ i.p.) treatments were performed 3, 10 and 14 days after tumour transplantation, again with a 24 h interval between the administration of DBD and CDDP for each sequence. The growth of the tumours was determined at the indicated time after transplantation by volume changes. The tumour volumes ($V$) were calculated according to the formula $V = \pi AB^2/6$, where $A$ is the greatest diameter and $B$ the diameter at peripendicular to $A$. The tumour volume at any time '$t$' ($V_t$) was compared with the volume of the inoculated tumour ($V_0$). Mean $V_t V_0$ (± s.d.) for each group was plotted as a function of time to obtain growth curves and the doubling time of the tumour growth calculated as described by Jones *et al.* (1990). In the spleen–liver system, the number and volume of the metastatic nodules were calculated after fixation of the liver in Dubosque–Brasil–Bouin (DBB) fixative 29 days after transplantation. Animals were weighed concurrently with tumour measurements and the mean weight (± s.d.) of each group expressed as the percentage change in the starting weight. In one experiment to reduce the renal toxic action of CDDP 0.5 ml of physiological saline was administered i.p. 30 min before and after CDDP treatment.

### *Cytotoxicity measurements on spheroids*

V79 Chinese hamster cells were grown in tissue culture as spheroids as previously described (Durand, 1986). For evaluating selected populations of viable cells from different depths within spheroids, a cell sorter-based technique was applied. Briefly, spheroids exposed to the test compounds for 1 h were stained by adding 2 μM Hoechst 33342 for 20 min prior to spheroid disaggregation. After removing excess drug and stain the spheroids were reduced to a single-cell population by using 0.25% trypsin (37°C, 10 min). Cell separation was accomplished with the use of a Becton Dickinson FACS 440, equipped with dual-argon lasers. Ten populations each comprising 10% of the cells were sorted on the basis of the slow penetration and rapid binding of the non-toxic fluorescent dye Hoechst 33342. The clonogenicity of each recovered cell population was determined, thus producing a 'chemosensitivity profile' through the entire spheroid (Durand, 1982, 1986). After the analysis of the effects of the two agent treatments, the observed and the expected survival curves were plotted as described previously (Durand, 1990).

### *Measurements on DNA damage*

The direct DNA-damaging action of the compounds was investigated by measuring the cleavability of plasmid DNA by restriction nucleases and also by the demonstration of interstrand cross-links (Calcuccia *et al.*, 1991; Hartley *et al.*, 1991). Bluescript M13 plasmid DNA (1 μg) in 20 μl of TEA buffer (70 mM TRIS + 2 mM EDTA adjusted to pH 8 with acetic acid) was incubated with the test compounds (dissolved in TEA buffer) at 37°C for 1 h. From the series of concentrations applied previously in these studies 0.3 μM CDDP and 3.3 μM DBD or its solvolytic products (DAD, BAD) were selected as most relevant in terms of plasma concentration and intermediate activity. Desalting and drug removal were performed by using an ultrafree MC Millipore filter, and then 2 units of restriction nuclease was added to cleave DNA at specific base sequences. *Bam*HI nuclease selectively cleaves G/GATCC, whereas the *Sma* nuclease acts on CCC/GGG and *Eco*RI on G/AATT base sequences. The optimum pH was found to be 7.4 and 6.8 for digestion with *Bam*HI and *Sma* respectively. After the 2 h digestion period the samples were mixed with the loading buffer (0.25% bromophenol blue, 40% sucrose, 50 mM EDTA, 40 mM Tris acetate pH 7.5). The restriction fragments were separated by horizontal electrophoresis using 0.8% (w/v) agarose gel containing 1 μg ml$^{-1}$ ethidium bromide in 40 mM Tris-acetate–1 mM EDTA (pH 8.0) at 80 V for 4 h. The changes in the mobility of the digested plasmid DNA indicated nuclease activity and the resulting fragments were expressed as a percentage of the supercoiled form. To determine interstrand cross-links, the bluescript M13 plasmid DNA was linearised by digestion with *Bam*HI and treated with the test compounds as described above. After removal of the test compounds by using an MC Millipore filter, the DNA samples were denatured (90°C for 2 min) and chilled in an ice-water bath prior to loading on 0.8% (w/v) agarose gels to separate single-and double-stranded DNA. This method was based on the one introduced by Hartley *et al.* (1991) with the exception that non-labelled DNA was used and changes in the percentage of the single-stranded DNA were recorded. The DNA bands were visualised under illumination from a short-wave UV light and were photographed with a Polaroid camera. The developed negatives were scanned with a UV–visible densitometer supported by software for densitogram analysis (Electrophoretic Data Center, Helena, USA).

### *Statistical analysis*

Significant differences between control and treatment (DBD, CDDP) values were determined using a two-tailed $t$-test. Differences between means giving a probability of less than 5% were considered as significant (i.e. $P<0.05$). The combination index (CI) for two drugs were calculated by using a multiple drug-effect analysis (program B) introduced by Chou and Talalay (1984) and kindly provided by Dr J Hoffman (University of Innsbruck, Austria). According to this analysis the CI is the ratio of the combination dose to the sum of the isoeffective single agent doses, consequently CI<1 indicates synergism and CI>1 indicates antagonism.

### Results

Mice bearing P388 solid lymphomas had a survival time of 12 ± 1.6 days after transplantation. Treatment at an advanced stage of tumour growth, i.e. 5 days after transplantation, with CDDP or DBD alone induced modest prolongation of the survival time (Figure 1). However the combined application of CDDP and DBD resulted in a further prolongation of the survival time compared with animals treated with either of the drugs alone. Figure 1 shows that, when using 7 mg kg$^{-1}$ CDDP, the survival time was especially augmented by both the low (80 mg kg$^{-1}$) and the high (750 mg kg$^{-1}$) doses of DBD. The combination index (CI) of Chou and Talalay (1984) varied with the doses of DBD. Analysing the effect of 80 mg kg$^{-1}$ DBD, the CI values were less than 1 if 3 or 7 mg kg$^{-1}$ CDDP was administered (0.78 and 0.23 respectively). It is noteworthy that 80 mg kg$^{-1}$ DBD with 1 or 9 mg kg$^{-1}$ CDDP resulted in antagonistic action (CI 1.2 and 1.9 respectively). On the other hand, administering 750 mg kg$^{-1}$ DBD jointly with 1, 3, 7 or 9 mg kg$^{-1}$ CDDP induced CI indexes indicating synergism (i.e. 0.8, 0.29, 0.05 and 0.69 respectively). In these mice there was no indication of increased lethality owing to toxicity; in fact 40% cure (alive 60 days after transplantation) was observed

**Figure 1** Anti-tumour activity of CDDP against P388 solid lymphomas after pretreatment with DBD. Mice were transplanted subcutaneously with $10^6$ P388 lymphoma cells then treated with DBD on day 5 and CDDP on day 6 after transplantation. Each group was composed of ten mice ($DBA_2 \times C57BL$, $F_1$ hybrid). Error bars ±s.d. ■, CDDP alone; □ CDDP + DBD 80 mg kg$^{-1}$; ●, CDDP + DBD 750 mg kg$^{-1}$.

after the administration of 750 mg $kg^{-1}$ DBD and 7 mg $kg^{-1}$ CDDP.

Figure 2 shows that tumour volumes for subcutaneous xenografts of HT168 human melanoma after the combined treatment with CDDP and DBD were significantly reduced.

The doubling time changes also indicated the greater antitumour action of this combination relative to CDDP or DBD alone; the value for the untreated tumour was 6.2 days, which was prolonged to 9.0, 13.1 and 20.7 days in the CDDP- DBD- and CDDP + DBD-treated experimental groups respectively.

In another series of experiments, the effects of this combination on metastasis formation from the same tumour transplanted into the spleen were investigated (Table I). Notably, the number of metastatic nodules was 86% higher after CDDP treatment compared with the control value. Most interestingly, this adverse response to CDDP was abolished if DBD was administered, especially if CDDP was given first and then DBD. Conversely, the total volume of all metastatic nodules per liver was a lower level in all drug-treated groups. This implies that, while the cellular growth rate in the metastatic nodules could be reduced, the actual number of tumour cells seeding the liver was unaffected by single-agent treatment. The observation that the combined application of CDDP followed by DBD reduced both the number and the volume of the metastatic nodules thus appears quite significant.

Unfortunately, the combined action of CDDP + DBD caused a significant reduction in body-weight (Figure 3). To avoid host toxicity the mice were hydrated 30 min before and 30 min after CDDP treatment by administering 0.5 ml of 0.9% sodium chloride. Figure 3 shows that no extreme loss of body weight was observed in the hydrated mice after CDDP + DBD treatment, indicating that toxic action of the combination could be reduced.

As in previous reports, the external cycling cells of the V79 spheroids were most sensitive to CDDP, with less cytotoxicity at greater depths within the spheroids (Figure 4). These studies provide the first indication that DBD action is qualitatively different from that of CDDP, as can be clearly seen from the differing sensitivity profiles in Figure 4. Interestingly, DBD decreased the clonogenic capacity of the internal cells more extensively than those cells of the external region, suggesting a potential role of hypoxia in modulating DBD activity. The combined application of DBD and CDDP resulted in more cytotoxicity; the response was apparently more than additive (Figure 4a and b).

As both DBD and CDDP have the capacity to bind and damage DNA, an obvious question is whether synergism can also be observed at the molecular level, or whether other

**Figure 2** Changes in the tumour volumes of HT-168 human melanomas after treatment with CDDP and DBD. There were ten CBA mice in each experimental group. Measurements of tumour size were performed as described in the Materials and methods section. □, Controls; ●, CDDP; O, DBD; ■, CDDP + DBD; ▲, DBD + CDDP.

**Figure 3** Changes in body weight of CBA immunosuppressed HT-168 melanoma-bearing mice after treatment with CDDP + DBD. Experimental groups were composed of ten mice as described in the Materials and methods section. □, Controls; ●, CDDP; O, DBD; Δ, CDDP + DBD; ▲, DBD + CDDP; ■, CDDP + DBD + 0.9% sodium chloride.

**Figure 4** Survival of spheroid cells exposed to CDDP and DBD, shown as a function of cell location in the spheroid at the time of treatment. **a**, □, CDDP 1 μg $ml^{-1}$; O, DBD 10 μg $ml^{-1}$; +, combination expected; ■, combination observed; **b**, □, CDDP 2 μg $ml^{-1}$; O, DBD 20 μg $ml^{-1}$; +, combination expected; ■, combination observed.

**Table I** Effect of DBD + CDDP combination on metastatic formation of HT-168 melanoma. Spleen-liver model evaluated at the 29th day after transplantation

| | *Control* | *CDDP* | *DBD* | *CDDP+DBD* | *DBD+CDDP* |
|---|---|---|---|---|---|
| Number of metastatic nodules and range | 6.61 ± 7.95 (0–25) | 12.33 ± 24.8 (0–17) | 6.51 ± 9.27 (0–29) | 3.29 ± 5.62 (0–11) | 6.29 ± 5.62 (1–16) |
| Volume of metastatic nodules ($mm^3$) | 39.64 ± 56.85 | 18.87 ± 26.9 | 25.27 ± 27.06 | 2.45 ± 3.73[a] | 10.61 ± 10.12 |
| Body weight (g) | 24.23 ± 2.0 | 21.81 ± 1.27 | 21.86 ± 2.61 | 17.12 ± 3.02 | 19.74 ± 1.71 |

Treatments were carried out as described in the Materials and methods. [a]Indicates significant change from control value.

**Table II** Modification of nuclease digestion of bluescript M13 plasmid DNA treatment with CDDP alone or in combination with DBD, DAD or BAD

| | *Cleaved plasmid DNA as a percentage of the supercoiled form* | |
|---|---|---|
| *Treatment* | Bam*HI digestion at pH 7.4* | Sma *digestion at pH 6.8* |
| Control[a] | 97.21±0.81 | 96.30±6.35 |
| CDDP (0.3 μM) | 58.95±5.69 | 47.97±4.79 |
| DBD (3.3 μM) | 88.22±3.71 | 86.35±5.02 |
| DAD (3.3 μM) | 79.81±3.80 | 54.00±2.96 |
| BAD (3.3 μM) | 101.40±1.70 | 96.65±0.63 |
| CDDP + DBD | 98.33±6.28 | 51.47±0.57 |
| CDDP + DAD | 95.86±1.83 | 60.94±1.06 |
| CDDP + BAD | 64.70±0.96 | 103.27±0.38 |

[a]Bluescript M13 plasmid DNA was digested with either *Bam*HI or *Sma* as described in the Materials and methods section. Data presented are from a representative experiment, mean±s.d. ($n$ = 2).

**Figure 5** Identification of cross-links and single-strand breaks of DNA. **a**, Separation of double- and single-stranded DNA on gel electrophoresis without drug treatment. The first peak shows double stranded DNA, the second peak represents the greater mobility of single-stranded DNA. **b**, The appearance of cross-linked DNA fragments at the position of double-stranded DNA after treatment with CDDP (0.3 μM) and heat denaturation. **c**, Appearance of single-strand breaks with smaller fragment size after DBD treatment (3.3 μM) and heat denaturation. **d**, Appearances of both cross-links and single-strand breaks of DNA after simultaneous treatment with CDDP + DBD and heat denaturation.

factors might contribute to the enhanced anti-tumour efficacy upon the combined application of these agents. Since cell-associated drug metabolism is not a prerequisite step for either CDDP or DBD action there seemed to be no objection to using naked DNA to investigate the combined action of these drugs. The cleavage of bluescript M13 plasmid DNA induced by *Bam*HI, *Sma* or *Eco*RI restriction nucleases was examined to detect drug binding at specific sites in the DNA. Digestion with nucleases resulted in a 96–97% conversion of the plasmid DNA from the supercoiled to the linearised form. This process was remarkably lower after treating the DNA with some of the test compounds. Treatment with 0.3 μM CDDP and 3.3μM DBD was shown to prevent cleavage of plasmid DNA by approximately 50% and 13% respectively regardless of whether *Bam*HI or *Sma* was used as the restriction nuclease. However, no changes occurred if DNA was cleaved by *Eco*RI in the presence of DBD (data not shown). Interestingly, DAD, the active solvolytic product of DBD, was more potent, especially if *Sma* was applied at pH 6.8. Conversely, BAD, the other solvolytic product of DBD, was completely inactive. The unique feature of the action DBD and its metabolites (DAD, BAD) was that in their presence there was no reduction in the nuclease activity induced by CDDP. By measuring *Bam*HI nuclease activity, an apparent antagonism was observed between CDDP + DBD and CDDP + BAD, whereas BAD affected only the inhibitory action of CDDP against the *Sma* digestion (Table II). Measurements of cross-link formation led to the conclusion that CDDP and DBD treatment induce different types of DNA damage, as it was shown that CDDP induces cross-linkage, whereas after DBD treatment formation of single strand breaks was the dominant change (Figure 5).

### Discussion

The results reported here suggest that the anti-tumour efficacy of DBD and cisplatin in combination is greater than the efficacy of a single application of either drug alone in a human metastatic tumour model and in mice bearing solid P388 lymphomas. In the latter model a remarkable increase in the survival time was also observed.

The biological changes induced by the combined application of DBD + CDDP showed certain qualitative differences from the action of either drug alone. This was apparent in the human metastatic model (HT-168 melanoma) in which DBD was not effective and CDDP increased the number and decreased the volume of the metastatic nodules (Table II). It is noteworthy that the combined use of the two drugs resulted in a significant reduction in both the number and volume of the metastatic nodules. This raised the question of whether greater anti-tumour efficacy of this combination could develop at a cell population or at a molecular level. Investigation using the V79 spheroids led to the conclusion that cells located at the external and internal regions of the spheroids show different sensitivity to DBD and CDDP. Studies on plasmid DNA showed that DBD treatment causes primarily single-strand breaks, whereas cross-links are formed after CDDP treatment. The two drugs acted synergistically in the production of strand breaks, but the number of CDDP-induced cross-links was reduced in the presence of DBD. Drug antagonism in this model system may be due to the solvolytic metabolites of DBD, because the mechanism of action of DAD and BAD in reversing the effect of CDDP was different in the *Bam*HI and *Sma* nuclease assays, and in the latter assay system DBD showed no interference in CDDP action. These findings may be an explanation for the dose-dependent synergism between DBD and CDDP if one considers the possibility that the ratio of the metabolites generated from DBD is dose dependent (Figure 1). The formation of BAD and DAD from DBD was characterised as solvolysis and both compounds were identified both in the plasma of patients treated with DBD and in a cell-free system (Horváth *et al*., 1979, 1982; Vidra *et al*., 1982; Kelley *et al*., 1986). It is conceivable that DBD + CDDP synergism may be determined by the type and concentration of DBD metabolites.

In the last few years, more emphasis has been placed on drug activity in hypoxic tumour cells, and several methods of killing hypoxic tumour cells are currently being investigated (Sartorelli, 1988). Numerous new chemical entities have been designed which have no reactive capacity unless they are activated by bioreductive processes under hypoxic conditions (Sartorelli, 1988; Walton *et al*., 1989). Often, the cytotoxic action of such agents can be augmented through manipulation of blood flow (Brown, 1991). Since DBD appears to be preferentially active against hypoxic cells in spheroids at therapeutically relevant doses, though with much lower oxygen-dependent specificity than many other bioreductive drugs, it may find application in combination with cisplatin as well as other drugs not effective against hypoxic cell subpopulations. Certainly the internal cells in the spheroids have other characteristic features, such as adaptation to the hypoxic surroundings. Enlargement of spheroids is accompanied by changes in ploidy and in extracellular matrix constituents and by a decrease in extracellular pH (Olive and Durand, 1994). Consequently, factors other than hypoxia could determine the higher efficacy of DBD against the cells located at the internal region of the spheroid.

In addition to the 'complementary' toxicity patterns for DBD and CDDP, our data also suggest synergistic interactions at the tumour, cellular and DNA levels. The marked increase in DNA strand break production by combination treatments is notable, as is the apparent 'antagonism' of the drugs in some cleavage assays. The latter results presumably indicate a qualitative difference in the molecular lesions formed, a result not incompatible with synergistic cytotoxicity. These observations clearly require additional study.

In summary, dibromodulcitol appears to be a promising new chemotherapeutic agent owing to its preferential toxicity towards hypoxic cells *in vitro*. This utility is amplified by the observed synergism with cisplatin, and suggests that DBD may also prove efficacious in combination with a number of other conventional cancer chemotherapy agents.

**Acknowledgements**
Supported by grants from the Hungarian National Scientific Research Fund (Grant Nos. T 006070 and 3024) and the Medical Research Council of Canada.

**References**

BELLET RE, CATALANO RB, MASTRANGELO MJ AND BERD D. (1978). Positive phase II trial of dibromodulcitol in patients with metastatic melanoma refractory to DTIC and a nitrosourea. *Cancer Treat. Rep.*, **62**, 2095-2099.

BROWN JM. (1991). Targeting bioreductive drugs to tumours: is it necessary to manipulate blood flow? *Int. J. Radiat. Biol.*, **60**, 231-236.

CALUCCIA M, FANIZZI FP, GIANNINI G, GIORDANO D, INTINI FP, LACIDOGNA G, LOSETO F, MARIGGIO MA, NASSI A AND NATILE G. (1991). Synthesis, mutagenicity binding to pBR 322 DNA and antitumour activity of platinum (II) complexes with ethambutol. *Anticancer Research*, **11**, 281-288.

CHOU TC AND TALALAY P. (1984). Quantitation analysis of dose-effect relationships: the combined effects of multiple drugs or enzyme inhibitors. *Adv. Enzyme Regul.*, **22**, 27-55.

DURAND RE. (1982). Use of Hoechst 33342 for cell selection from multicell systems. *J. Histochem. Cytochem.*, **30**, 117-122.

DURAND RE. (1986). Chemosensitivity testing in V79 spheroids: drug delivery and cellular microenvironment. *J. Natl Cancer Inst.*, **77**, 247-252.

DURAND RE. (1990). Cisplatin and CCNU synergism in spheroid cell subpopulations. *Br. J. Cancer*, **62**, 947-953.

HARTLEY SA, BERARDINI MD AND SOUHAMI RL. (1991). An agarose gel method for the determination of DNA interstrand crosslinking applicable to the measurement of the rate of total and second arm cross-links reactions. *Anal. Biochem.*, **193**, 131-134.

HORVÁTH IP, CSETÉNYI S, KERPEL-FRONIUS S, HINDY I AND ECKHARDT S. (1979). Metabolism and pharmacokinetics of dibromodulcitol (DBD, NSC-104800) in man. I. metabolites of DBD. *Eur. J. Cancer*, **15**, 337-344.

HORVÁTH IP, SOMFAI-RELLE S, HEGEDÜS L AND JARMAN M. (1982). Toxicity, antitumour and haematological effects of 1,-anhydro-6-bromogalacticol and D-mannitol: a comparison with the related dibromo- and dianhydro-derivatives. *Eur. J. Cancer Clin. Oncol.*, **18**, 573-577.

INSTITORIS E AND TAMAS J. (1983). Identification of guanine and adenine adducts in DNA alkylated by dibromodulcitol *in vitro* and *in vivo*. *Chem. Biol. Interact.*, **47**, 133-144.

JENEY A, DZURILLAY É, LAPIS K AND VALKÓ É. (1979). Chromatin proteins as a possible target for antitumour agents. Alterations of chromatin proteins in dibromodulcitol treated Yoshida tumours. *Chem. Biol. Interactions*, **26**, 349-361.

JONES AL, MILLAR SC, POWELL BCB, SELBY P, WINKLEY A, LAKHANI S, GORE ME AND McELWAIN TS. (1990). Enhanced antitumour activity of carmustine (BCNU) with tumour necrosis factor *in vitro* and *in vivo*. *Br. J. Cancer*, **62**, 776-780.

KELLEY S, PETERS PW, ANDERSEN J, FURLONG EA, FREI E AND HENNER WD. (1986). Pharmacokinetics of dibromodulcitol in humans: a Phase II study. *J. Clin. Oncol.*, **4(5)**, 753-761.

LADÁNYI A, TIMÁR J, PAKU S, MOLNÁR G AND LAPIS K. (1990). Selection and characterization of human melanoma lines with different liver-colonizing capacity. *Int. J. Cancer*, **40**, 456-461.

OLIVE PL AND DURAND RE. (1994). Drug and radiation resistance in spheroids cell contact and kinetics. *Cancer Metastasis Rev.*, **13**, 121-138.

SARTORELLI AC. (1988). Therapeutic attack of hypoxic cells of solid tumors: presidential address. *Cancer Res.*, **48**, 775-778.

STEHMAN FB, BLESSING JA, McGEHEE R AND BARRETT RJ. (1989). A phase II evaluation of dibromodulcitol (NSC 104800) in patients with advanced squamous cell carcinoma of the cervix. a Gynecologic Oncology Group study. *J. Clin. Onc.*, **7**, 1892-1895.

VIDRA I, SIMON K, INSTITORIS I, CSÖREGH I AND CZUGLER M. (1982). The chemical transformation products of 1,6-dibromo-1,6 dideoxygalacticol and 1,2-5,6-dianhydrogalacticol in aqueous solution. *Carbohydr. Res.*, **111**, 41-57.

WALTON MI, WOLF CR AND WORKMAN P. (1989). Molecular enzymology of the reductive bioactivation of hypoxic cell cytotoxins. *Int. J. Radiat. Oncol. Biol. Phys.*, **16**, 983-986.